# American Heart Association’s Cholesterol CarePlan as a Smartphone-Delivered Web App for Patients Prescribed Cholesterol-Lowering Medication: Protocol for an Observational Feasibility Study

**DOI:** 10.2196/resprot.9017

**Published:** 2019-01-24

**Authors:** Maria Woringer, Kanika I Dharmayat, Geva Greenfield, Alex Bottle, Kausik K Ray

**Affiliations:** 1 Imperial College London London United Kingdom

**Keywords:** behavioral change, cholesterol, lifestyle, mHealth, medication adherence

## Abstract

**Background:**

Adoption of healthy lifestyle and compliance with cholesterol-lowering medication reduces the risk of cardiovascular disease (CVD). The use of digital tools and mobile technology may be important for sustaining positive behavioral change.

**Objective:**

The primary objective of this study is to evaluate the feasibility and acceptability of administering the Cholesterol CarePlan Web app developed by the American Heart Association aimed at improving lifestyle and medication adherence among patients prescribed cholesterol-lowering medication. The secondary objective is to assess the Web app’s efficacy.

**Methods:**

A prospective, observational feasibility study will be conducted to demonstrate whether the Web app may be successfully taken up by patients and will be associated with improved clinical and behavioral outcomes. The study will aim to recruit 180 study participants being prescribed cholesterol-lowering medication for at least 30 days across 14 general practices in London, England. Potentially eligible patients will be invited to use the Web app on a smartphone and visit general practice for three 20-minute clinical assessments of blood pressure, height, weight, smoking, and nonfasting cholesterol over 24 weeks. The feasibility of administering the Web app will be judged by recruitment and dropout statistics and the sociodemographic and comorbidity profile of consenting study participants, consenting nonparticipants, and all potentially eligible patients. Acceptability will be assessed using patients’ readiness to embrace new technologies, the usability of the Web app, and patient satisfaction. The efficacy of the Web app will be assessed by changes in medication adherence and clinical risk factors by levels of the Web app compliance.

**Results:**

This study is currently funded by the American Heart Association. Initial study recruitment will take place between February and July 2018 followed by patient follow-up. Patient level data will be obtained in January 2019. Data analysis will be completed by February 2019. Results will be submitted for publication in March 2019.

**Conclusions:**

The potential of an app to improve patients’ lifestyle and management of cholesterol may inform the design of a randomized controlled trial and the delivery of more effective CVD prevention programs.

**International Registered Report Identifier (IRRID):**

PRR1-10.2196/9017

## Introduction

### Background

Cardiovascular disease (CVD) is a major cause of disability and premature mortality worldwide accounting for a third of deaths annually in England [1,2]. CVD contributes considerably to the rising cost of health care and is estimated to cost the National Health Service (NHS) and the United Kingdom economy £30 billion annually [1]. Much of the CVD burden is largely preventable [3-5]. Behavioral modification in relation to physical activity, nutrition, smoking, and alcohol consumption, as well as adherence to cholesterol-lowering and blood pressure- lowering medication, is associated with a reduction in the risk of CVD [5]. Prescribing statins is important for the primary prevention of CVD [6-11]. With a lower threshold for statin prescription in the United Kingdom and the United States, many more people will be prescribed lipid-lowering drugs [12-14]. Despite statin therapy, many individuals remain at risk of CVD events owing to behavioral factors such as poor medication adherence and adoption of healthy lifestyles [15]. A combined approach to control cholesterol should include better medication adherence and changes to lifestyle.

Primary CVD prevention programs are increasingly delivered using digital tools and mobile technology [16]. Used in health promotion, digital interventions may enable patient-centered care with improved communication, greater responsiveness to patient needs, and shared decision making [17,18]. Research showed digital interventions to be effective in improving cholesterol levels, medication adherence, weight loss, physical activity, smoking cessation, hypertension, and self-management of diabetes mellitus [16,19,20]. Such research was limited to short message services (SMS) text messages. In contrast, smartphones may enable accessing sophisticated content, such as self-monitoring programs and educational videos, to help patients monitor their risk factors and educate themselves to improve lifestyle and medication adherence. Growing smartphone ownership in the United Kingdom in the adult population makes smartphones an attractive platform for health interventions [21]. Nonetheless, a lack of scientifically designed and tested interventions impedes the successful primary prevention of CVD [22]. Of more than 43,000 apps listed in the health and fitness category of Apple’s iTunes Store in 2014, almost half were misclassified or were loosely affiliated with health and fitness [22]. Of 710 cardiology-related apps available in 2013, very few were designed for CVD prevention [22]. In addition, few health-related apps were developed per evidence-based guidelines and had quantifiable benefit for improving clinical outcomes [22]. Health-related apps are not being rigorously evaluated for the acceptability, sustainability of engagement, and the impact on clinical risk factors [16]. Good compliance with smartphone apps was described as content completion ranging from 69% to 72% [23-25]. Reportedly, declining engagement and attrition are the main barriers to the efficacy of digital health interventions [16,26].

The American Heart Association (AHA), a US charity devoted to CVD prevention, developed the Cholesterol CarePlan Web app for patients prescribed a cholesterol-lowering medication. This Web app delivered to patients on their smartphones through the internet aims to increase patients’ awareness of the benefits associated with compliance with medication, regular physical activity, and a healthy diet while monitoring these behavioral risk factors over time. The Cholesterol CarePlan is a 12-week care plan for self-management of cholesterol focusing on lifestyle and medication compliance, with weekly reminders and educational videos designed using evidence-based guidelines such as AHA’s CVD risk, cholesterol, and lifestyle guidelines [13,27,28]. Using 7 health factors and lifestyle behaviors that support heart health, it is based on the AHA’s “ideal cardiovascular health.” These factors and behaviors, called “Life’s Simple 7,” are smoking status, healthy diet, physical activity, healthy weight, blood pressure, cholesterol, and blood glucose. Improvements in these 7 areas can increase the quality of life and lifespan [29]. The Web app is simple to use, comes from credibly sourced information, contains educational information including benefits of behavioral change, real-time tracking of biometric data, and may be self-administered by patients in consultation with their health care providers. The feasibility, acceptability, and efficacy of the AHA Cholesterol CarePlan have not been assessed among patients.

### Aims and Objectives

The main aim of this work is to establish whether the AHA Cholesterol CarePlan may be administered to patients as a Web app to improve cholesterol management and lifestyle among patients prescribed a cholesterol-lowering medication. The primary study objectives are to evaluate the feasibility and acceptability of the AHA Cholesterol CarePlan delivered as a Web app. The secondary objectives are to evaluate the Web app’s efficacy.

## Methods

### Study Design

This is a prospective, observational feasibility study involving the use of a Web app on a smartphone for 24 weeks along with the collection of clinical measurements in general practice at baseline and 12 and 24 weeks after the Web app administration.

### Sample Size

This study will aim to recruit 180 patients to participate in the study and come for clinical assessments. A formal power calculation for the sample size was not conducted as this project was funded as a feasibility study. Therefore, sample size estimates are not required.

### Inclusion Criteria

Adults aged >18 years being prescribed cholesterol-lowering medication for established vascular disease, diabetes, familial hypercholesterolemia, or high-risk primary prevention for at least 30 days prior to being invited to take part in the study will be included. Cholesterol-lowering medication includes statins (atorvastatin, pravastatin, rosuvastatin, and simvastatin), ezetimibe, aspirin, clopidogrel, and colesevelam. In addition, patients must own any Apple or Android smartphone with access to the internet, give informed consent, and provide 3 nonfasting blood samples.

### Exclusion Criteria

Patients not currently being prescribed cholesterol-lowering medication or being prescribed for <30 days prior to being invited to take part in the study will be excluded. In addition, patients not owning a smartphone, unable to consent to research, and having insufficient command over English will be excluded. The capacity of participants will be monitored by a health care professional during 3 clinic visits. Individuals who lose capacity during the course of the study will also be excluded.

### Setting and Site Selection

The National Institute for Health Research (NIHR) Clinical Research Network North West London will contact research active practices with the goal of setting up to 14 general practices for research across North West London. Research active practices are defined as practices with a previous record of carrying out research activities.

### Participant Enrollment

To participate in the study, potentially eligible patients will be identified from the general practice databases by administrative staff at each participating general practice using automated eligibility search pertaining to prescribed medication. To maximize recruitment, patients will be invited by post, SMS text messaging, and opportunistically. General practices using post and text invitations will invite patients to come to prescheduled appointments. Practices inviting patients opportunistically will invite patients as part of routine clinical care. Patients will be invited in the order of acceptance until 180 patients come into their first visit.

Patients invited by post will be mailed study materials (invitation and patient information sheet) by general practice administrative staff. Opportunistically recruited patients will be invited by their health care professionals and receive study materials in paper copy. Finally, patients invited by SMS text messaging on their mobile phones by general practice administrative staff will receive study materials electronically. Patients who decide not to participate will be given an option to complete an anonymous sociodemographic survey either in paper copy with a prepaid envelope (if invited by post or opportunistically) or using a weblink to a Web-based Qualtrics survey (if invited by SMS text messaging). There will be no financial incentives for study participation.

### Data Collection

The sociodemographic survey containing information on comorbidities (stroke, heart disease, hypertension, familial hypercholesterolemia, kidney disease, type 2 diabetes, and dementia), age, gender, ethnicity, education, partial postcode, and smartphone ownership will be collected among study participants through the Web app. In addition, sociodemographic data (not including smartphone ownership or education) for study participants will be obtained from their medical records by general practices. Among consenting nonparticipants, the sociodemographic survey will be administered anonymously (with no possibility of further data linkage) either using a paper or a Web-based survey. All potentially eligible patients will not be asked to respond to the sociodemographic survey. Instead, anonymous sociodemographic data will be obtained from these patients’ medical records by general practices. The number of invited individuals will be recorded by the general practice staff. Medication adherence at weeks 1, 12, and 24 will be collected from secondary data.

During the baseline visit, health care professionals will set up participants to use the Web app from their work computer with the internet using a prespecified Web address on a clinician interface of the Web app. During 20-minute consultations at weeks 1, 12, and 24, a health care professional will take and input clinical measurements (blood pressure, height, weight, smoking, and nonfasting cholesterol) into the clinician interface of the Web app (see Multimedia Appendix 1).

The Web app will send out an invitation to patients’ smartphone. Patients will be asked to log in and respond to the sociodemographic survey during baseline visit and medication adherence questions during 3 clinic visits. All other questions will be completed outside clinic. The AHA Cholesterol CarePlan consists of 12 weekly components. This includes Life Simple 7 questions; 12 weekly questionnaires pertaining to diet, physical activity, and taking of medication; 12 self-reported weight and blood pressure readings; 9 educational videos; and a Patient Satisfaction Survey. This is a total of 76 multiple choice questions (46 unique questions) and 9 videos (6 unique videos). In addition, the following questionnaires will be administered through the Web app: sociodemographics, Technology Readiness Index (TRI) [30], System Usability Scale (SUS) [31], Medication Adherence Rating Scale (MARS) [32], and Short-Form Health Survey (SF-12) [33]. This is a total of 99 multiple choice questions (55 unique questions). It should take a patient 5-15 minutes to complete questions on a weekly basis. Patients will have a week to answer each week’s questions and may resume at a later time. Patients will be sent electronic reminders to complete weekly questions and book follow-up consultations (see Multimedia Appendix 2).

No equipment will be provided for self-monitoring of blood pressure and weight, and these questions will be optional. Multimedia Appendix 3 shows a timeline of quantified assessments recorded through the Web app.

Among patients who may choose not to complete the CarePlan, the questionnaires including sociodemographics, TRI, SUS, MARS, SF-12, and Patient Satisfaction will be administered through a Web-based Qualtrics survey after 12 weeks. These patients will be asked to complete sociodemographic and MARS in clinic and all other questions outside clinic. In addition, the MARS and SF-12 will be administered after 24 weeks through a Web-based Qualtrics survey. Patients will be asked to complete the MARS in clinic and the SF-12 outside clinic. Patients will have a week to complete these questions.

### Data Handling and Security

Data collected through the Web app, including responses to weekly questionnaires, clinical measurements (cholesterol, blood pressure, weight, and self-reported smoking), and personal data (including the name, date of birth, and mobile number), will be hosted securely by the Digital Healthcare Management (DHM) in partnership with UKCloud. Upon study completion, the host will deidentify data and transfer anonymous data to Imperial College London (ICL). In addition to the data collected through the Web app, ICL will collect secondary data pertaining to prescribed medication among study participants directly from general practices. Furthermore, ICL will analyze all data and send data in aggregate form to the AHA (see Multimedia Appendix 4).

To eliminate the potential breaches of confidentiality, all necessary measures were taken to ensure the data are safe and that all data captured electronically are only transferred in an anonymized form. The DHM will act as the data host that will initialize and run the Web app. The DHM is a provider of a complete end-to-end patient health monitoring system using mobile consumer electronics and is a preferred partner of UKCloud. The DHM will use UKCloud’s infrastructure to store the data collected through the Web app. UKCloud (formerly Skyscape Cloud Services) Enterprise Compute Cloud provides a trusted, connected, and flexible cloud platform for critical enterprise apps. It is Pan-Government Accredited by the National Cyber Security Centre from UK government accreditors. In addition, it is an N3 (the trusted broadband network for NHS healthcare) aggregator that has been approved by NHS Digital to allow NHS and non-NHS organizations to host apps and data on its cloud that connect to the N3 and transmit or store Patient Identifiable Data. UKCloud manages the external firewalls that connect to the internet and other government networks. In addition, it uses trend micro for antivirus, malware, and vulnerability scanning purposes. UKCloud notifies customers of breaches to the firewall. Data stored on UKCloud are backed up daily. The data will be stored in accordance with Vivify’s BYOD (Bring Your Own Device) and Caregiver Portal requirements. VivifyHealth is a digital health platform designed to streamline remote care. Confidentiality of the data will be maintained by the DHM and ICL through password protection of the data and stored in secure servers at ICL following a transfer from the DHM. Moreover, survey data collected via Qualtrics, a survey provider available by subscription to Imperial College London, will be accessible with a password by the study team and will not contain any personal data.

### Statistical Analysis

The feasibility of administering the AHA Cholesterol CarePlan will be judged by recruitment and dropout statistics as well as by the sociodemographic and comorbidity profile of study participants in comparison with those of invited patients and all eligible patients. The participation rate will be judged as the percentage of people who consent to take part in the study out of all invited patients. General descriptive statistics will describe the sociodemographics and comorbidities of (1) consenting study participants; (2) consenting nonparticipants; and (3) all potentially eligible study patients. Invited patients will be described in terms of self-reported smartphone ownership. This will inform the likely generalizability of subsequent findings.

Compliance with the Web app will be measured using the frequency and duration of use. Patients’ weekly participation in the Web app will be automatically tracked, as well as whether they download educational videos. Compliance with the Web app will be measured using the number of weekly questionnaires attempted, the number of questions completed, and the number of educational videos downloaded. Furthermore, compliance will be described as a percentage of content accessed out of all content presented through the Web app throughout the 12-week care plan, as well as on a weekly basis.

It is anticipated that patients may be grouped into full compliers if they complete 12 weeks, good compliers if they complete 8 weeks, poor compliers if they complete 1-7 weeks, and noncompliers if they do not complete any of the weekly AHA Cholesterol CarePlan questions. Among compliers with the Web app, medication persistence and changes in diet and physical exercise will be examined throughout the 12-week Cholesterol CarePlan, and changes in the quality of life, self-reported medication adherence will be assessed at weeks 1, 12, and 24. In addition, the self-reported quality of life will be assessed using the Short-Form Health Survey [33]. The self-reported behavioral change in relation to diet and physical exercise, as well as medication persistence, will be assessed through weekly questions in the AHA Cholesterol CarePlan. Furthermore, self-reported adherence to cholesterol- lowering medication will be examined using the MARS [32].

The acceptability of the Cholesterol CarePlan will be assessed using patients’ readiness to embrace new technologies, the usability of the Web app, and patient satisfaction. Patients’ behavior in relation to readiness to adopt new technology will be examined using the 12-item TRI [30]. The Web app usability will be assessed using the 10-item SUS [31]. Furthermore, patient satisfaction with the Cholesterol CarePlan will be assessed using a questionnaire developed by the AHA.

The efficacy of the Cholesterol CarePlan will be judged by examining the Web app compliance with changes in medication adherence and clinical risk factors. Medication adherence will be collected from secondary data using the Medication Possession Ratio and Proportion of Days Covered. These measures are based on the number of days medication supplied and the quantity of medication dispensed for each filled prescription [34,35]. A difference-in-difference (DID) analysis using multiple linear regression, controlling for education, socioeconomic deprivation, ethnicity, gender, and age will be performed. Using DID, a time-series average change in medication adherence, cholesterol, smoking, blood pressure, and body mass index over 3 time-points will be compared among full compliers and partial compliers, respectively, with Web app noncompliers. Interactions in regression models will be explored using explanatory variables. In addition, subgroups analyses (by education, socioeconomic deprivation, ethnicity, gender, and age) will explore differences in medication adherence and clinical outcomes among full compliers, partial compliers, and noncompliers with the Web app. Finally, using multiple regression modeling while controlling for education, socioeconomic deprivation, ethnicity, gender and age, average changes in diet, physical exercise, and quality of life will be examined among Web app compliers.

As this is a feasibility study, the anticipated effects are neither known in terms of the prespecified compliance level with the Web app nor in terms of the expected reduction in behavioural risk factors or clinical outcomes. The feasibility study will assess possible effects in the first place. The number of invited participants from the potentially eligible population group will be monitored each week with the invitations sent out dependent upon the response rate and the achievement of this sample size. The feasibility will be evaluated through the invitations sent out and the number agreeing to participate each week. This feasibility study will inform us of the possibility of obtaining a sample of study participants among the potentially eligible population for a larger trial. For the same reason, we will not adjust *P* value thresholds for multiple testing; statistical significance will be judged using the *P*<.05 threshold.

### Ethical Considerations

Prior to being awarded the funding grant, the study underwent AHA’s internal medical review examining its medical importance, design, quality, and safety reporting. In addition, the study underwent external peer review, received favorable ethics opinion from the North of Scotland Research Ethics Committee, Health Research Authority approval, and was adopted onto the NIHR Clinical Research Network portfolio.

## Results

This study is currently funded by the American Heart Association. Initial study recruitment will take place between February and July 2018 followed by patient follow-up. Patient level data will be obtained in January 2019. Data analysis will be completed by February 2019. Results will be submitted for publication in March 2019.

## Discussion

To the best of our knowledge, this is the first study examining the feasibility and acceptability of administering AHA’s Cholesterol CarePlan as a Web app to patients. The benefits to patients of using the AHA Cholesterol CarePlan may be improved medication adherence, changes to diet, physical exercise, clinical risk factors, and quality of life. The benefits to the scientific community include an understanding of whether the Web app may be successfully taken up by patients and whether it may help improve lifestyle and management of cholesterol. The benefits to health services may be the delivery of more effective CVD prevention programs.

Although patients treated for hyperlipidemia with cholesterol- lowering drugs will be aware of their CVD risk and the need for cholesterol-lowering medication, as well as lifestyle changes, they may not adhere to medication, engage in a regular physical activity, or consume a healthy diet, all of which are needed for successful cholesterol management. The action and maintenance of behavioral change may require ongoing motivational support. The maintenance of behavioral change in relation to medication adherence and lifestyle is important for controlling cholesterol and preventing the premature onset of CVD [12,14,36]. To change patients’ behavior to prevent the premature onset of CVD, according to the Health Belief Model, patients must perceive themselves to be at increased risk of CVD, perceive the severity of illness, and weigh the barriers to behavioral change against the benefits thereof [37]. An important factor for controlling cholesterol, according to the Transtheoretical Model, is the maintenance of behavioral change in relation to medication adherence and lifestyle [38].

Persuasive technology using convincing and effective communication is theorized to help change behavior in relation to CVD. This technology is modeled on ideas that behaviors change only when people are motivated, able and have appropriate triggers [22]. Interventions delivered through smartphones may improve medication adherence [36]. Given the high level of penetration of smartphones among people of lower socioeconomic status, there is an opportunity for health-related mobile apps to overcome traditional barriers to CVD prevention. Nonetheless, a possible limitation of using smartphones for the prevention of CVD is the existence of the digital divide with lower socioeconomic groups retaining older technologies. An important barrier to app use is privacy concerns in relation to data sharing and personal safety. Despite observed associations between app use and positive behavioral change, there is limited evidence on the sustainability of behavioral change using apps [22].

A possible methodological threat to study design may be in selection bias with more wealthy patients owning a smartphone being able to participate in the study; we will be able to assess to what extent those who take part in our study represent the wider eligible population. Another possible limitation may be the use of self-reported questionnaires. However, the use of biometric assessments and medication prescribing may cross-validate the self-reported medication compliance. As this is a feasibility study, no sample size calculations were required. Once study effects are established, the aim is to apply for further funding to design a randomized controlled trial with fully specified sample size criteria.

## Appendix

Multimedia Appendix 1 Data collected by the Web app.

Multimedia Appendix 2 Patient pathway.

Multimedia Appendix 3 The timeline of quantified assessments recorded through the Web app.

Multimedia Appendix 4 Data flow diagram.

## Abbreviations

AHA: American Heart Association
CVD: cardiovascular disease
DHM: Digital Healthcare Management
DID: difference-in-difference
ICL: Imperial College London
MARS: Medication Adherence Rating Scale
NHS: National Health Service
NIHR: National Institute for Health Research
N3: the trusted broadband network for NHS healthcare
SF-12: Short-Form Health Survey
SMS: short message services
SUS: System Usability Scale
TRI: Technology Readiness Index
