# Supplementary material for: American Heart Association’s Cholesterol CarePlan as a Smartphone-Delivered Web App for Patients Prescribed Cholesterol-Lowering Medication: Protocol for an Observational Feasibility Study
Source: JMIR Res Protoc. 2019 Jan 24;8(1):e9017. doi: 10.2196/resprot.9017 (PMC6365873; doi:10.2196/resprot.9017)
Supplement: Multimedia Appendix 1 [file resprot_v8i1e9017_app1.pptx]

## Slide 1
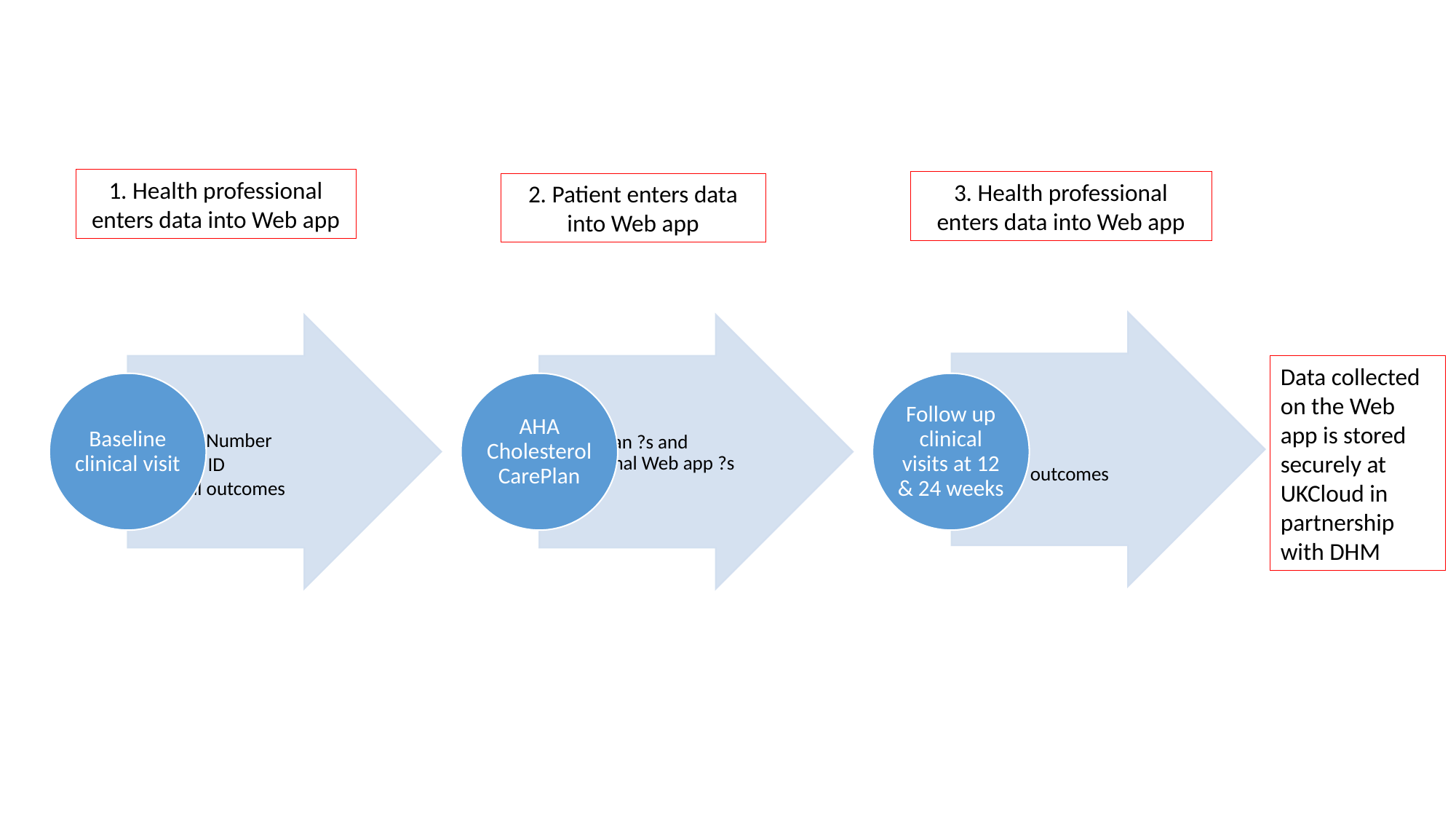

1. Health professional enters data into Web app
3. Health professional enters data into Web app
2. Patient enters data into Web app
Data collected on the Web app is stored securely at UKCloud in partnership with DHM
