# Supplementary material for: American Heart Association’s Cholesterol CarePlan as a Smartphone-Delivered Web App for Patients Prescribed Cholesterol-Lowering Medication: Protocol for an Observational Feasibility Study
Source: JMIR Res Protoc. 2019 Jan 24;8(1):e9017. doi: 10.2196/resprot.9017 (PMC6365873; doi:10.2196/resprot.9017)
Supplement: Multimedia Appendix 2 [file resprot_v8i1e9017_app2.pptx]

## Slide 1
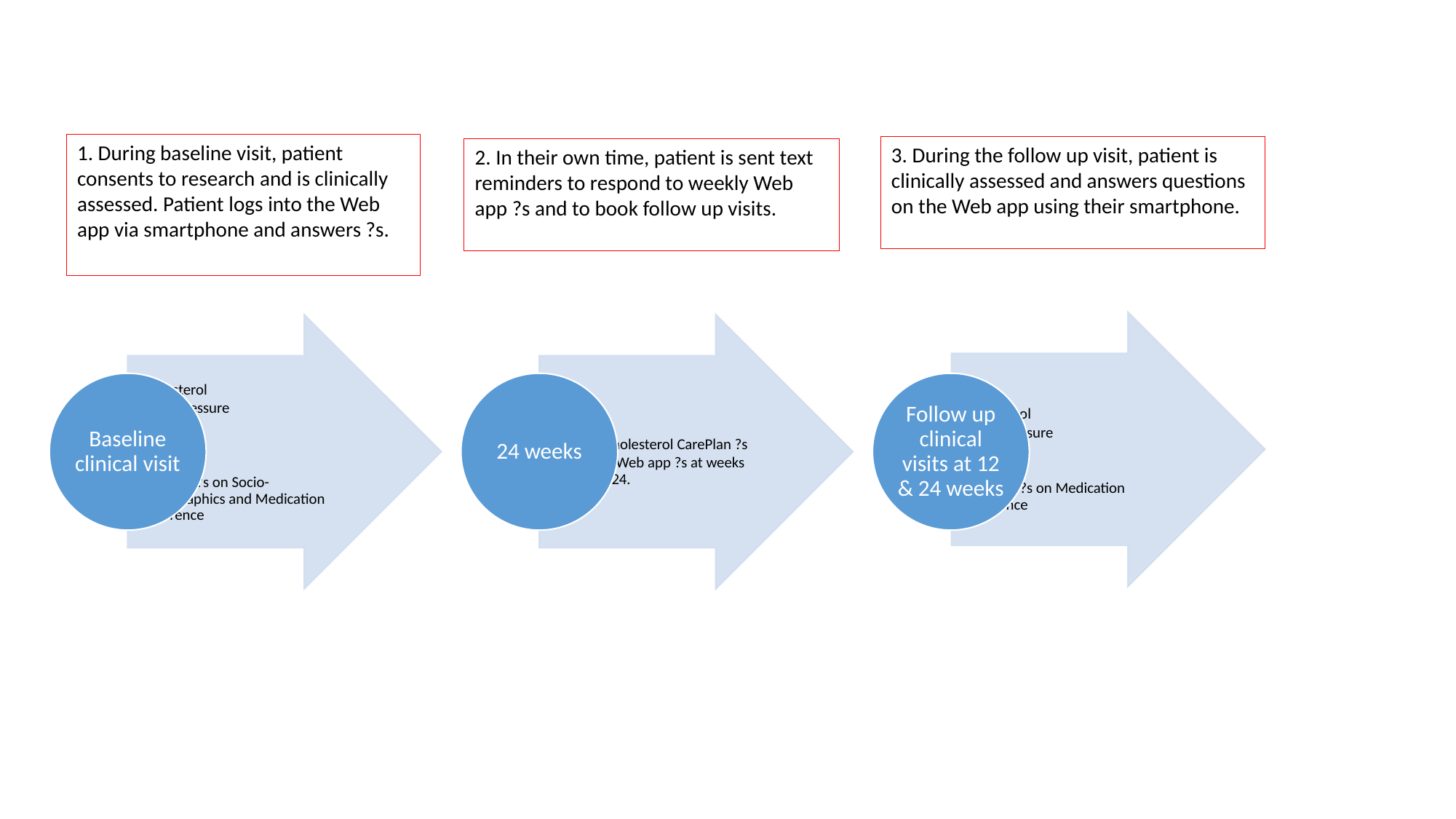

1. During baseline visit, patient consents to research and is clinically assessed. Patient logs into the Web app via smartphone and answers ?s.
3. During the follow up visit, patient is clinically assessed and answers questions on the Web app using their smartphone.
2. In their own time, patient is sent text reminders to respond to weekly Web app ?s and to book follow up visits.
