# Supplementary material for: American Heart Association’s Cholesterol CarePlan as a Smartphone-Delivered Web App for Patients Prescribed Cholesterol-Lowering Medication: Protocol for an Observational Feasibility Study
Source: JMIR Res Protoc. 2019 Jan 24;8(1):e9017. doi: 10.2196/resprot.9017 (PMC6365873; doi:10.2196/resprot.9017)
Supplement: Multimedia Appendix 4 [file resprot_v8i1e9017_app4.pptx]

## Slide 1
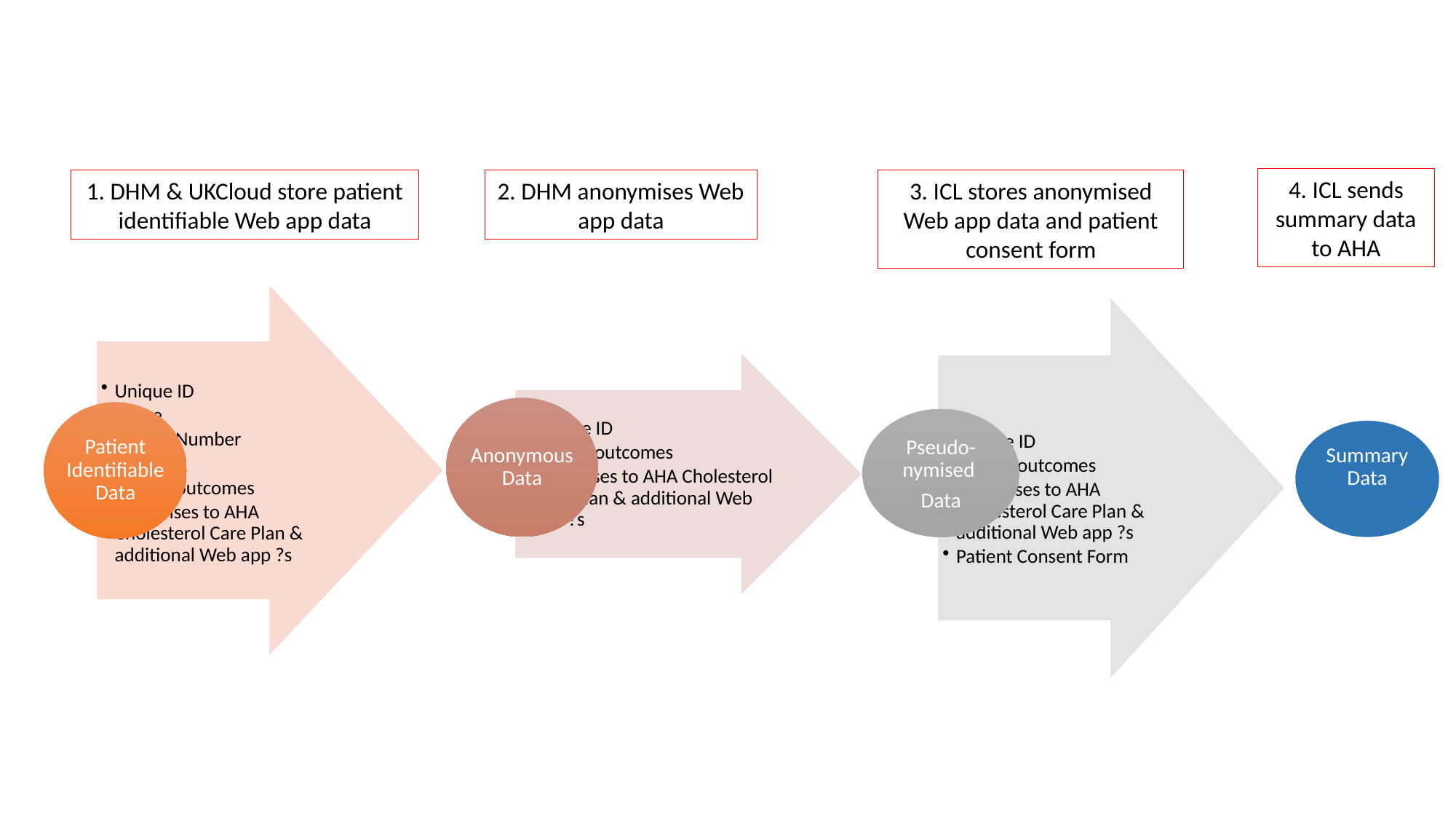

4. ICL sends summary data to AHA
1. DHM & UKCloud store patient identifiable Web app data
2. DHM anonymises Web app data
3. ICL stores anonymised Web app data and patient consent form
Summary Data
